# Regulation of PACAP receptors in respiratory distress syndrome (RDS) of preterms and newborns

**DOI:** 10.1186/2194-7791-2-S1-A19

**Published:** 2015-07-01

**Authors:** MJ Müller, A Kislat, A Hippe, A Poppe, S Goldmann, T Paul, S Seeliger

**Affiliations:** Department of Pediatric Cardiology, Intensive Care Medicine and Neonatology, Georg August University Medical Center, D-37075 Göttingen, Germany; Department of Dermatology, Heinrich Heine University, D-40225 Düsseldorf, Germany; Children´s Hospital, D-86633 Neuburg/Donau, Germany

## Background

Prevalence of respiratory distress syndrome (RDS) caused by primary surfactant deficiency reaches 50% in infants below 32 weeks of gestational age. Increased alveolar surface tension leads to atelectatic and/or dystelectatic lung areas followed by inflammation resulting in development of a bronchopulmonary dysplasia (BPD) in 8% of these children. Standard RDS treatment consists of administration of exogenous surfactant. With the introduction of less invasive application (LISA) of exogenous surfactant, the use of Surfactant has significantly increased. Pituitary adenylate cyclase-activating polypeptide (PACAP) and its receptors (VPAC1, VPAC2, PAC1) trigger inflammatory reactions as well as anti-inflammatory processes.

## Materials and methods

Using cell cultures, FACS and ELISA methods we analyzed surfactant effects on inflammation and PACAP-regulation and of its receptors in vivo and ex vivo.

## Results

We were able to show that lipopolysaccharide (LPS) in prestimulated cells (PBMC) after surfactant treatment resulted in a significant up-regulation of pro-inflammatory interleukin (IL)-8. In tracheal secretory cells of surfactant-treated neonates we found an up-regulation of pro-inflammatory receptor VPAC1. Furthermore, in cell cultures we identified changes in the proliferation rates. Amniotic infection syndrome (AIS) is one of the major causes for premature birth resulting in RDS requiring exogenous surfactant. After pre-stimulation of PBMCs and subsequent addition of surfactant, pro-inflammatory signals were fortified. These results were confirmed by analysis of tracheal secretions of preterms and newborns.

## Conclusions

Current findings may imply that safety and efficacy of routine surfactant administration should be reassessed as surfactant potentially triggers pulmonary inflammation and thus may contribute to BPD development.

